# Diversity and Antibiograms of Bacterial Organisms Isolated from Samples of Household Drinking-water Consumed by HIV-positive Individuals in Rural Settings, South Africa

**DOI:** 10.3329/jhpn.v30i3.12286

**Published:** 2012-09

**Authors:** A. Samie, M.B. Mashao, P.O. Bessong, T.F. NKgau, M.N.B. Momba, C.L. Obi

**Affiliations:** ^1^Department of Microbiology, University of Venda, Private Bag X5050, Thohoyandou 0950, Limpopo, South Africa; ^2^Department of Environmental, Water and Earth Sciences, Tshwane University of Technology, Arcadia Campus, Private Bag X 680, Pretoria 0001, South Africa; ^3^Academic and Research Division, Walter Sisulu University, Mthatha, South Africa

**Keywords:** Antibiotic resistance, Bacteria, Diarrhoea, Drinking-water, HIV, Opportunistic infections, Water quality, South Africa

## Abstract

Diarrhoea is a hallmark of HIV infections in developing countries, and many diarrhoea-causing agents are often transmitted through water. The objective of the study was to determine the diversity and antibiotic susceptibility profiles of bacterial organisms isolated from samples of household drinking-water consumed by HIV-infected and AIDS patients. In the present study, household water stored for use by HIV-positive patients was tested for microbial quality, and isolated bacterial organisms were analyzed for their susceptibility profiles against 25 different antibiotics. The microbial quality of water was generally poor, and about 58% of water samples (n=270) were contaminated with faecal coliforms, with counts varying from 2 colony-forming unit (CFU)/100 mL to 2.4×10^4^ CFU/100 mL. Values of total coliform counts ranged from 17 CFU/100 mL to 7.9×10^5^/100 mL. In total, 37 different bacterial species were isolated, and the major isolates included *Acinetobacter lwoffii* (7.5%), *Enterobacter cloacae* (7.5%), *Shigella* spp. (14.2%), *Yersinia enterocolitica* (6.7%), and *Pseudomonas* spp. (16.3%). No *Vibrio cholerae* could be isolated; however, *V. fluvialis* was isolated from three water samples. The isolated organisms were highly resistant to cefazolin (83.5%), cefoxitin (69.2%), ampicillin (66.4%), and cefuroxime (66.2%). Intermediate resistance was observed against gentamicin (10.6%), cefepime (13.4%), ceftriaxone (27.6%), and cefotaxime (29.9%). Levofloxacin (0.7%), ceftazidime (2.2%), meropenem (3%), and ciprofloxacin (3.7%) were the most active antibiotics against all the microorganisms, with all recording less than 5% resistance. Multiple drug resistance was very common, and 78% of the organisms were resistant to three or more antibiotics. Education on treatment of household water is advised for HIV-positive patients, and measures should be taken to improve point-of-use water treatment as immunosuppressed individuals would be more susceptible to opportunistic infections.

## INTRODUCTION

Acquired immunodeficiency syndrome (AIDS), first described in 1981, has become the most devastating global epidemic the world has ever faced. At the end of 2008, an estimated 60 million people, globally, were infected with HIV. More than five million people were newly infected each year, and more than 6,000 died every day due to AIDS (1). HIV, the causative agent of AIDS, infects humans and destroys their immune systems, making them more susceptible to opportunistic organisms, the majority of which are found in the environment in which we live. Water is a basic need for all living organisms, including humans. However, water is also a vector for different organisms, including viruses, bacteria, fungi, and parasites (2). Many organisms transmitted by water cause diarrhoea, which is a very common symptom among HIV-infected individuals. Approximately 90% of individuals infected with HIV have suffered from chronic diarrhoea, thereby making it a common cause of morbidity and mortality among HIV-infected and AIDS patients (3). The link between HIV and water and sanitation is, thus, unavoidable, and the understanding of the intricacies between HIV and AIDS and water is still to be clarified. The vulnerability of HIV-positive individuals to organisms transmitted through water has been demonstrated all over the world (4,5). Results of studies in Uganda indicated higher degrees of contamination in household drinking-water consumed by HIV-infected and AIDS patients and demonstrated that the implementation of the safe water system among persons with HIV was significantly associated with a 25% reduction in diarrhoea episodes and 33% fewer days with diarrhoea (6).

Predominantly rural and poverty-stricken communities around the Venda areas of the Limpopo province of South Africa still do not have access to safe, clean drinking-water on a daily basis. Results of studies conducted in this region showed that residents often collect water from contaminated river sources (7,8). Water from these river sources is used for various purposes, including drinking, bathing, and washing and are also often used for domestic animals (9). Municipal taps provided in the regions often go for days and, in some cases, for weeks without water. Only a few residents have boreholes in their households leaving others to rely on alternative sources, such as the river. Collected water is often stored in big containers for weeks before use, and the condition in which the water is stored often predisposes it to contamination (10). Contaminated water, poor sanitation, and poor personal hygiene are the main causes of enteric diseases. Enteric diseases affect everyone; however, the diseases are more severe in HIV-positive individuals because of their weakened immune system (2).

Results of studies around the world showed that diarrhoeal diseases can be decreased by treating drinking-water before use, and such treatment methods include boiling the water or adding a quantified amount of bleach to the water before use (11,12). However, poor rural people do not have access to such vital information while others choose to ignore it as they have been using the water for some time. When afflicted by diseases, residents seek treatment, and antibiotics are prescribed but the variable nature of infecting organisms and their antibiotic resistance do limit the choice of antibiotics, thereby making such antibiogram studies a necessity (13). Regular updates on isolates obtained from household drinking-water and their antibiotic susceptibility may assist the health officials in combating diarrhoea among HIV-positive and AIDS patients.

Despite the high prevalence of HIV infections in South Africa and the high prevalence of diarrhoeal diseases, there is a paucity of updated information on the diversity of potential bacterial pathogens that could cause diarrhoea among HIV-infected people. In the present study, samples of water, stored for consumption in the households with HIV-infected and AIDS patients in rural areas of the Limpopo province, were analyzed to determine their microbiological quality and bacteriological and antibiotic susceptibility profiles of isolated organisms. Such information may be vital in the control and management of waterborne infections in the era of HIV/AIDS.

## MATERIALS AND METHODS

### Study population and sample collection

HIV-infected and AIDS patients living in the Makhado Municipality of the Limpopo province were invited to participate in the study. The objectives of the study were clearly explained to the potential participants. Their rights to withdraw from the study were also explained to them. Each participant who agreed to participate in the study was required to sign a consent form. Both females and males participated in the study. Once the individuals agreed to participate in the study, samples of water were collected from the storage containers in their homes in sterile 500-mL bottles and transported to the laboratory within two hours after collection.

### HIV testing

The HIV status of the individuals was tested according to the testing strategy II of the Joint United Nations Programme on HIV and AIDS/World Health Organization (14), and a non-linked anonymous approach was used. However, pre- and post-test counselling was provided to volunteers who wanted to know their results, and their samples were appropriately coded to enable the tracing of patients. All the study subjects were screened for antibodies to HIV with the OraQuick HIV1/2 kit (OraSure Technologies, USA), using oral fluids. Screening for HIV seroprevalence was performed as recommended by the manufacturers of the kit. These screening tests have accepted sensitivities and specificities for HIV antibody screening surveys (15).

### Determination of general quality of household water

The samples collected were transported on ice to the Microbiol­ogy Laboratory of the University of Venda for microbiological assays. Microbiological parameters, such as heterotrophic plate counts, total coliform counts, and faecal coliform counts, were determined using standard microbiological methods (14). The membrane filtration method was used for all counts. Plate count agar, m-Endo agar, and m-FC agar were used for hetero­trophic counts, total coliform counts (TCs), and faecal coliform counts (FCs) respec­tively. Each test was done in triplicate, and the geometric means were recorded.

The organisms used as control were *Escherichia coli* (ATCC 8739), *Proteus vulgaris* (ATCC 6830), *Pesudomonas aeruginosa* (ATCC 7700), *Serratia marsecens* (ATCC 9986), and *Streptococcus epidermidis* (ATCC 29212).

### Isolation of bacterial organisms

The membrane filtration method as described under total microbial quality was employed. However, the filters were placed aseptically on thiosulphate-citrate-bile salts-sucrose (TCBS) agar, *Campylobacter* blood-free agar, *Salmonella-Shigella* agar and m-Endo agar to isolate *Vibrio*, *Campylobacter*, *Salmonella/Shigella*, and *E. coli* respectively. Incubation for *Campylobacter* was in microphillic condition at 44.5 ºC while the other organisms were grown under aerobic condition at 37 ºC for 24-48 hours (16). Subculturing was performed to obtain pure colonies by streaking into fresh plates. Pure colonies were incubated at 37 ºC for 24 hours. After 24 hours of incubation, nutrient broth was prepared in MacCartney bottles. Pure colonies were stored in slant in MacCartney bottles and stored in the fridge for preservation. Presumptive identification of colonies was also done based on growth characteristics. Different biochemical tests, including Gram-staining, oxidase, catalase, gas production, and sugar fermentation, were conducted for preliminary identification.

### Identification of bacteria and determination of antibiogram

Subculturing was performed to obtain pure colonies from MacCartney bottles on fresh plates. The identity of cultures and their sensitivities were determined using the MicroScan autoscan analyzer (Dade Behring, Inc., West Sacramento, CA) following instructions of the manufacturer (17). The antibiotic susceptibility profiles of the organisms were determined using the broth micro-dilution method using the combo panels from MicroScan, and the results were analyzed as described by the Clinical and Laboratory Standards Institute (18). The antibiotics used included: amikacin, amoxicillin/clavulate, ampicillin, aztreonam, cefazolin, cefepime, cefotaxime, cefotaxime/k clav, cefoxitin, ceftazidime, ceftazidime/k clav, ceftriaxone, cefuroxime, ciprofloxacin, ertapenem, gentamicin, imipenem, levofloxacin, meropenem, piperacillin, piperacillin/tazobactam, tetracycline, ticarcillin/clavulanic acid, tobramycin, trimethoprim/sulpha-methoxazole, and ampicillin/sulbactam.

### Statistical analysis

All the data were entered into an Excel sheet and uploaded onto the SPSS software (version 17.2). Statistical analysis was conducted using the chi-square test on the SPSS software (version 17.2).

### Ethical clearance

The Ethical Committee of the University of Venda approved the study protocol. Authorization to collect samples was obtained from the Department of Health of the Limpopo province. Due to the stigmatization of the disease, the study team chose to work closely with support groups, non-governmental organizations (NGOs), and HIV care-givers because they provide a ‘comfort zone’ for HIV-infected/AIDS patients, who, in turn, confide in the care-givers. Before the commencement of the study, members of the research team made preliminary visits to each of the chosen study areas. During these visits, the background, protocols, objectives, and potential significance of the study, including issues around confidentiality and consent were discussed with care-givers, support groups, and NGOs, and their support was sought for the collection of specimens. The support groups, care-givers, and NGOs worked directly with HIV-infected/AIDS patients, which provided the platform to reach out to the participants.

## RESULTS

### Demographic characteristics of study population

In total, 270 HIV-positive patients were recruited to participate in the study. Two hundred seventy water samples were collected from different households with HIV-positive and AIDS patients. Of those patients whose demographic data were known, 63 (23.3%) were male, and 90 (33.3%) were female. The age of the individuals varied from 1 to 81 years, with 32% of the individuals aged 20-39 years. Table 1 shows the demographic information of the study population.

### General microbial quality of household drinking-water

The microbial quality of the collected water samples was determined using heterotrophic plate count (HPC), FC, and TC. The results for HPC ranged from 42 colony-forming unit (CFU)/100 mL to 8.4×10^8^ CFU/100 mL. The results for TC ranged from 17 CFU/100 mL to 7.9×10^5^ CFU/100 mL while the results for FC ranged from 2 CFU/100 mL to 2.4×10^4^ CFU/100 mL. Table 2 shows the results of the microbial quality of the collected water samples.

**Table. 1.** Demographic information of study population

Parameter	Characteristics	No.	%
Gender	Male	63	23.3
	Female	90	33.3
	Unknown	117	43.3
Age-group (years)	0-9	4	1.5
	>9-19	18	6.7
	>19-29	40	14.8
	>29-39	39	14.4
	>39-49	19	7.0
	>49-59	9	3.3
	>59-81	14	5.2
	Unknown	143	53.0

**Table. 2.** Microbiological characteristics of drinking-water samples collected from households with HIV-infected/AIDS patients in rural areas of Vhembe district

Indicator	Minimum	Maximum	Mean	Median
Heterotrophic plate counts	42	8.4×10^8^	1.1×10^7^	2.2×10^3^
Total coliforms	17	7.9×10^5^	2.7×10^5^	6.1×10^2^
Faecal coliforms	2	2.4×10^4^	2.1×10^3^	74

### Profile of bacterial organisms isolated

From the 270 water samples collected, 134 organisms were isolated comprising 37 different bacterial species. The most common isolates included: *Pseudomonas* spp. (16.3%), *Shigella* spp. (14.2%), *Acinetobacter lwoffii* (7.5%), *Enterobacter cloacae* (7.5%), Y*ersinia enterocolitica* (6.7%), and *E. coli* (6.0%). Table 3 shows the frequency of different organisms isolated.

### Antibiotic susceptibility profiles of isolated organisms

Twenty-five different antibiotics were used for determining the antibiotic susceptibility profiles of the organisms isolated. The results obtained were analyzed according to the CLSI standards, and the strains were further classified as resistant, indeterminate, or sensitive. There was a high level of resistance of the organisms to cefazolin (83.5%), cefoxitin (69.2%), ampicillin (66.4%), and cefuroxime (66.2%). Intermediate resistance was observed against gentamicin (10.6%), cefepime (13.4%), ceftriaxone (27.6%), and cefotaxime (29.9%). Levo-floxacin (0.7%), ceftazidime (2.2%), meropenem (3%), and ciprofloxacin (3.7%) were the most active antibiotics against all the microorganisms, with all recording less than 5% resistance. The results are summarized in Table 4.

### Multiple drug resistance profiles of isolated organisms

Multiple drug resistance profiles defined as resistance to three or more antibiotics in this study were also observed. Of the 134 organisms isolated, 108 (80.6%) were multiple drug-resistant. The resistance ranged from three to 20 antibiotics at a time, with higher resistance to 13 (10.4%), followed by 9, 4, and 3 (8.2%) antibiotics at a time. None of the isolates was resistant to all the 25 antibiotics used while 18 (13.4%) were susceptible to all the antibiotics used. *Ralstonia paucula* had the highest rate of multiple drug resistance and was resistant to 20 antibiotics at a time, followed by *Chromobacterium violaceum* which was resistant to 18 antibiotics. Table 5 shows the distribution of multiple drug resistance, including the names of organisms resistant to the designated number of antibiotics.

## DISCUSSION

Water is one of the most important basic needs for living beings. It is often regarded as the source of life. However, the very same water may be a source of infections, particularly among immunocompromised patients, such as those infected with HIV and AIDS (12). Contaminated water and poor personal hygiene are the most important modes of transmission of enteric organisms. People residing in poor rural areas often collect water from surrounding rivers. The present study has demonstrated a high prevalence and diversity of enteric bacterial organisms in household water consumed by HIV-positive patients in the Limpopo province of South Africa.

Results of studies in the Vhembe region of South Africa showed that water sources used by the people were contaminated with potential enteric bacterial and viral pathogens (19). However, some of these residents do not have alternative water sources; hence, they continue using the water regardless of the risks involved. Furthermore, the level of hygiene in these populations is generally low (20). Water is regarded as clean and safe for consumption when it complies with the South Africa guidelines for drinking-water. These guidelines are used around the world, and when drinking-water does not comply with such guidelines, it is regarded as not suitable for human use. In this study, the HPCs, TCS, and FCs were used for analyzing the microbial quality of water. The results obtained indicate that the microbial quality of water was poor and not suitable for human consumption. These findings were similar to those previously obtained where water collected from various river sources around the Venda region was analyzed (7). The difference in the years in which the studies were conducted indicates that nothing has been done about the water sources used by the population. Measures to help the communities could include education and educational campaigns on household treatment of water. Results of studies indicate the importance of providing good-quality drinking-water to HIV-infected and AIDS patients (5,21). The poor quality of water may cause a wide range of diseases, including cholera, typhoid fever, hepatitis, dysentery, giardiasis, and other gastrointestinal infections in rural communities. The scope of bacterial organisms isolated in this study was broad, with *Pseudomonas* spp., *Shigella* spp., *A. lwoffii*, *E. cloacae*, *Y. enterocolitica,* and *E. coli* accounting for most isolates. *Shigella* organisms were the most commonly-isolated organisms in water samples in the present study. Recent studies have shown that these organisms were responsible for outbreaks of diarrhoea in an urban area in India (22). In a study in Nigeria, the most commonly-isolated organisms from water sources included *E. coli* (22.7%), *Enterobacter aerogenes* (2.5%), *Salmonella* spp. (13.3%), *Shigella* spp. (19.3%), *Proteus* spp. (18.5%), *Klebsiella* spp. (19.3%), and *P. aeruginosa* (4.2%) (23). *Shigella* has been identified as one of the most common pathogens responsible for the outbreaks of diarrhoea in Italy (24). Obi *et al*. reported the presence of *E. coli, V. cholerae, A. hydrophila, Shigella, Plesiomonas,* and *Campylobacter* spp. as the most common isolates in their study (9). The results obtained in this study, compared to those obtained elsewhere, indicate that the diversity of bacterial organisms isolated from drinking-water varies widely. Such diversity may have been brought on by the differences in the collected water samples and the laboratory techniques used for identifying the bacterial organisms.

**Table. 3.** Frequencies of bacterial species isolated from household water samples

Organism	Frequency (%)	Organism	Frequency (%)
*Achromobacter xylosoxidans*	2 (1.5)	*Proteus mirabilis*	2 (1.5)
*Acinetobacter lwoffii*	10 (7.5)	*Providencia rettgeri*	1 (0.7)
*Aeromonas hydrophila*	1 (0.7)	*Providencia stuartii*	1 (0.7)
*Alcaligenes* species	3 (2.2)	*Pseudomonas aeruginosa*	8 (6.0)
*Burkholderia cepacia*	2 (1.5)	*Pseudomonas fluorescens*	9 (6.7)
*Cedecea neteri*	1 (0.7)	*Pseudomonas luteola*	1 (0.7)
*Chromobacterium violaceum*	5 (3.7)	*Pseudomonas* species	1 (0.7)
*Citrobacter freundii*	1 (0.7)	*Pseudomonas stutzeri*	3 (2.2)
*Delftia acidovorans*	2 (1.5)	*Ralstonia paucula*	1 (0.7)
*Enterobacter agglomerans*	1 (0.7)	*Ralstonia pickettii*	1 (0.7)
*Enterobacter amnigenus*	5 (3.7)	*Raoultella ornithinolytica*	6 (4.5)
*Enterobacter cloacae*	10 (7.5)	*Serratia liquefaciens*	3 (2.2)
*Enterobacter sakazakii*	2 (1.5)	*Shewanella putrefaciens*	1 (0.7)
*Escherichia coli*	8 (6.0)	*Shigella* species	19 (14.2)
*Klebsiella oxytoca*	4 (3.0)	*Stenotrophomonas maltophilia*	2 (1.5)
*Klebsiella rhinoscleromatis*	1 (0.7)	*Tatumella ptyseos*	2 (1.5)
*Klebsiella* species	1 (0.7)	*Vibrio fluvialis*	3 (2.2)
*Leminorella* species	1 (0.7)	*Yersinia enterocolitica*	9 (6.7)
*Actinobacillus* species	1 (0.7)	Total	134 (100)

In the present study, organisms such as *Acinetobacter* were commonly isolated. The *Acinetobacter* genus consists of more than 30 species, of which *A. baumannii* and, to a lesser extent, genomic species 3 and 13 are mostly associated with the clinical environment and nosocomial infections (25). These organisms have not been reported from previous studies in the area and could be responsible for potentially fatal infections. In a recent study by Toyoshima and colleagues, *A. lwoffii* was found responsible for a fatal respiratory illness in a 68-year old female (26). Other studies have also reported on the emergence of *Acinetobacter* species other than *A. baumannii* which were often associated with bacteraemia (27). The detection of these organisms in water samples from HIV-infected patients in the Limpopo province indicates the possibility of involvement of these organisms in the pathogenicity of opportunistic infections among patients. In fact, *A. lwoffii* is known to be involved in several diseases, such as sepsis, bacteraemia, pyrexia, meningitis, post-haemorrhagic hydrocephalus, rigours, pneumonia, cellulitis, rash, ophthalmia neonatum, urinary tract infection (UTI), and abscess (28). Further studies are needed to identify the potential of this organism as an opportunistic pathogen among HIV-infected patients in the region. Results of a study in Italy indicate that *Acinetobacter* spp. were responsible for several diseases in HIV-infected and AIDS patients, including sepsis, UTIs, respiratory tract diseases, and bacteraemic pneumonia (29). In that study, the organisms responsible included *A. calcoaceticus*, *A. lwoffii*, and other *Acinetobacter* spp.

**Table. 4.** Overall activities of different antibiotics used against isolates

Antibiotic	Resistance No. (%)	Sensitive No. (%)	Indeterminate No. (%)	MIC range
Amikacin	17 (12.7)	117 (87.3)	0	4->32
Amox/k clav	76 (56.7)	43 (32.1)	15 (11.2)	≤8/4->16/8
Ampicillin	89 (66.4)	33 (24.6)	12 (9)	≤8->16
Aztreonam	53 (39.6)	65 (48.5)	16 (11.9)	≤4->16
Cefazolin	111 (83.5)	17 (12.8)	5 (3.8)	≤8->16
Cefepime	18 (13.4)	112 (83.6)	4 (3)	≤2->16
Cefotaxime	40 (29.9)	67 (50)	27 (20.1)	≤2->32
Cefotaxime/k clav	66 (49.3)	53 (39.5)	15 (11.2)	≤0.5->4
Cefoxitin	92 (69.2)	37 (27.8)	4 (3)	≤8->16
Ceftazidime	3 (2.2)	126 (94)	5 (3.7)	≤1->16
Ceftazidime/k clav	47 (35.1)	54 (40.3)	33 (24.6)	≤0.25->2
Ceftriaxone	37 (27.6)	70 (52.2)	27 (20.1)	≤4->32
Cefuroxime	88 (66.2)	40 (30.1)	5 (3.8)	≤4->16
Ciprofloxacin	5 (3.7)	125 (93.3)	4 (3)	≤0.5->2
Ertapenem	32 (24.2)	82 (62.1)	18 (13.6)	≤2->4
Gentamicin	14 (10.6)	108 (81.8)	10 (7.6)	≤1->8
Imipenem	7 (5.2)	121 (90.3)	6 (4.5)	≤1->8
Levofloxacin	1 (0.7)	129 (96.3)	4 (3.6)	≤2->4
Meropenem	4 (3)	123 (93.2)	5 (3.8)	≤1->8
Piperacillin	18 (13.7)	109 (83.2)	4 (3.1)	≤16->64
Pip/tazo	10 (8.2)	110 (90.2)	2 (1.6)	≤16->64
Tetracycline	26 (20.6)	92 (73)	8 (6.3)	≤4->8
Ticar/k clav	45 (33.6)	81 (60.4)	8 (6)	≤16->64
Tobramycin	10 (7.5)	118 (88.7)	5 (3.8)	≤1->8
Trimeth/sulpha	53 (39.6)	81 (60.4)	0	≤2/38->2/38
Amp/sulbactam	0	2 (66.7)	1 (33.3)	≤8/4->16/8

Amox=Amoxicillin;

Amp=Ampicillin;

Clav=Clavulate;

MIC=Minimum inhibitory contentration;

Pip=Piperacillin;

Sulpha=Sulphamethoxasole;

Tazo: Tazobactam;

Ticar=Ticarcillin;

Trimeth=Trime-thoprim

Antibiotic resistance constitutes an important setback in the control of bacterial infections. We found high resistance of the bacterial isolates to antibiotics, such as cefazolin, cefoxitin, and ampicillin. Results of recent studies in Pakistan indicate that about 57.5% of isolates from drinking-water were resistant to ampicillin while 42.2% were resistant to kanamycin (30). Resistance to ciprofloxacin was low in the present study (3.7%); however, this constituted an increase from 0% described in 2002 by Obi and Bessong but it was lower compared to 10% observed by Obi *et al*. (9,13). This resistance level is lower compared to studies conducted in Michigan, USA, where the level of resistance of coliforms to ciprofloxacin was 9.9% in drinking-water from tap-water samples in the city (31). However, we found a resistance level of 20.6% while, in Michigan, the resistance level to the same antibiotic was 3.8%. The level of resistance to gentamicin was 10.6% in the present study while Obi *et al*. found the resistance levels varying from 0% to 8% depending on the origin of the organisms tested.

The antibiotic susceptibility of the isolated organisms was also determined as such information is vital in the treatment and management of diarrhoeal diseases. It is also important as organisms have been shown to develop resistance against commonly-used antibiotics (32). Multiple drug resistance defined as resistance to three or more antibiotics was common, with 80.6% of the organisms exhibiting multiple drug resistance. These findings are similar to those obtained by Obi *et al*. who showed that bacterial isolates demonstrated multiple drug resistance to antibiotics (7,9). In the present study, we found a very low susceptibility to ceftriaxone. This antibiotic has been reported to be very potent. In a study in India, there was no evidence of resistance against this antibiotic, particularly among *Shigella* isolates from stool samples (33). Only 10% of the *Shigella* organisms in the present study were susceptible to this antibiotic. This is a cause for concern as ceftriaxone is one of the most recent antibiotics and is supposed to be used as the last resort. Such high resistance could be due to the overuse of this antibiotic in the hospital environment, leading to adaptive resistance. In a recent study of the isolates from stool samples in the Limpopo province, the resistance level varied from 8% to 15% depending on the organisms tested, with lower resistance among *Shigella* spp. (8%) (34). In a study in Iran, lower resistance rates were found among *Shigella* organisms (35).

**Table. 5.** Multiple drug resistance among organisms isolated from water samples

No. of antibiotics	No. of organisms	Organism
3	11	*Klebsiella* spp.; *E. sakazakii*^2^; *A. lwoffii*^2^; *Alcaligenes* spp.; *R*. *ornithinolytica*; *V. fluvialis*; *E. coli*; *K. oxytocoa*^2^
4	11	*E. cloacae*; *Y. Enterolitica*; *E. amnigenus*^3^; *A. lwoffii*; *R. ornithinolytica*^3^; *C. freundii*; *K. oxytoca*
5	6	*R. pickettii*; *E. cloacae*^2^; *P. stutzeri*; *Y. enterocolitica*; *R. ornithinolytica*
6	9	*Pseudomanas* spp.2; *Shigella* spp.; *Y. enterocolitica*; *B. cepacia*; *E. cloacae*^2^; *S. liquefaciens*^2^
7	8	*Pseudomonas* spp.2; *Shigella spp.*; *E. amnigenus*; *Alcaligenes* spp.; *Y. enterocolitica*; *E. cloacae*; *S. liquefaciens*
8	5	*D. acidovorans*; *A. lwoffii*; *Shigella* spp.; *E. cloacae^2^*
9	11	*V. fluvialis*; *P. fluorescens*; *Shigella* spp.^5^; *Alcaligenes* spp.; *A. xylosoxidans*; *P. fluorescens*^2^
10	8	*P. aeruginosa*^4^; *Shigella* spp.2; *P. fluorescens*; *A. hydrophila*
11	9	*P. aeruginosa^2^*; *Y. enterocolitica; Shigella* spp.3; *P. luteola*; *P. fluorescens*; *A. lwoffii*
12	8	*T. ptyseos; P. aeruginosa; Shigella* spp.3; *K. rhinoscleromatis*; *Y. enterocolitica*; *P. fluorescens*
13	14	*P. stuartii*; *Shigella* spp.2; *Leminorella* spp.; *A. xylosoxidans*; *C. violaceum*^3^; *A. lwoffii; V. fluvialis*; *P. aeruginosa*; *P. fluorecens*^2^; *B. cepacia*
14	4	*S. maltophillia*; *Y. enterocolitica*; *Shigella* spp.; *C. neteri*
15	1	*S. maltophillia*
16	1	*C. violaceum*
18	1	*C. violaceum*
20	1	*R. paucula*

Superscripts indicate the number of times the organism occurs in the same group

Profiles of resistance to about 20 antibiotics at a time were noted. In a study in Nigeria, Oluyege *et al*. found that over 10% of bacteria were resistant to four or more antibiotics, and antibiotic resistance was the highest in members of the genera *Enterobacter, Pseudomonas*, and *Proteus* (22). Similar results have also been reported in Pakistan and India (36,37).

The findings of the present study suggest that the water samples stored for consumption were contaminated with potential bacterial pathogens, and this poses a serious public-health threat as children and HIV-positive patients used such stored water daily. Education and educational campaigns on water storage, personal and environmental hygiene, and household treatment of water in rural communities are needed in this era of HIV/AIDS for improved healthcare delivery.

## ACKNOWLEDGEMENTS

The authors are grateful to the National Research Foundation, Water Research Commission, the University of Venda, and the Walter Sisulu University for financial support or provision of facilities.

## References

[1] Joint United Nations Programme on HIV/AIDS (2009). AIDS epidemic update: November 2009.

[2] Becker ML, Cohen CR, Cheang M, Washington RG, Blanchard JF, Moses S (2007). Diarrheal disease among HIV-infected adults in Karnataka, India: evaluation of risk factors and etiology. Am J Trop Med Hyg.

[3] Garcia C, Chincha O, Leon M, Iglesias D, Barletta F, Mercado E (2010). High frequency of diarrheagenic *Escherichia coli* in human immunodeficiency virus (HIV) patients with and without diarrhea in Lima, Peru. Am J Trop Med Hyg.

[4] Beatty GW (2010). Diarrhea in patients infected with HIV presenting to the emergency department. Emerg Med Clin North Am.

[5] Obi CL, Onabolu B, Momba MNB, Igumbor JO, Ramalivahna J, Bessong PO (2006). The interesting cross-paths of HIV/AIDS and water in Southern Africa with special reference to South Africa. Water SA.

[6] Lule JR, Mermin J, Ekwaru JP, Malamba S, Downing R, Ransom R (2005). Effect of home-based water chlorination and safe storage on diarrhea among persons with human immunodeficiency virus in Uganda. Am J Trop Med Hyg.

[7] Obi CL, Potgieter N, Bessong PO, Matsaung G (2002). Assessment of the microbial quality of river water sources in rural Venda communities in South Africa. Water SA.

[8] Obi CL, Potgieter N, Bessong PO, Igumbor EO, Green E (2003). Prevalence of pathogenic bacteria and rotaviruses in stools of patients presenting with diarrhoea from rural communities in Venda, South Africa. S Afr J Sci.

[9] Obi CL, Bessong PO, Momba MNB, Potgieter N, Samie A, Igumbor EO (2004). Profiles of antibiotic susceptibilities of bacterial isolates and physico-chemical quality of water supply in rural Venda communities, South Africa. Water SA.

[10] Clasen TF, Bastable A (2003). Faecal contamination of drinking water during collection and household storage: the need to extend protection to the point of use. J Water Health.

[11] Ngwenya BN, Kgathi DL (2006). HIV/AIDS and access to water: a case study of home-based care in Ngamiland, Botswana. Phys Chem Earth.

[12] Arvelo W, Kim A, Creek T, Legwaila K, Puhr N, Johnston S (2010). Case-control study to determine risk factors for diarrhea among children during a large outbreak in a country with a high prevalence of HIV infection. Int J Infect Dis.

[13] Obi CL, Bessong PO (2002). Diarrhoeagenic bacterial pathogens in HIV-positive patients with diarrhoea in rural communities of Limpopo province, South Africa. J Health Popul Nutr.

[14] World Health Organization (2007). Guidance on provider-initiated HIV testing and counselling in health facilities.

[15] Donovan BJ, Rublein JC, Leone PA, Pilcher CD (2004). HIV infection: point-of-care testing. Ann Pharmacother.

[16] Alam SI, Agarwal GS, Kamboj DV, Rai GP, Singh L (2003). Detection of spores of *Bacillus anthracis* from environment using polymerase chain reaction. Indian J Exp Biol.

[17] Bulik CC, Fauntleroy KA, Jenkins SG, Abuali M, LaBombardi VJ, Nicolau DP (2010). Comparison of meropenem MICs and susceptibilities for carbapenemase-producing *Klebsiella pneumoniae* isolates by various testing methods. J Clin Microbiol.

[18] Clinical and Laboratory Standards Institute (2006). Performance standards for antimicrobial susceptibility testing; sixteenth informational supplement.

[19] Obi CL, Momba MNB, Samie A, Igumbor JO, Green E, Musie E (2007). Microbiological, physico-chemical and management parameters impinging on the efficiency of small water treatment plants in the Limpopo and Mpumalanga provinces of South Africa. Water SA.

[20] Potgieter N, Obi CL, Bessong PO, Igumbor EO, Samie A, Nengobela R (2005). Bacterial contamination of Vhuswa—a local weaning food and stored drinking-water in impoverished households in the Venda region of South Africa. J Health Popul Nutr.

[21] Hall J, Hodgson G, Kerr KG (2004). Provision of safe potable water for immunocompromised patients in hospital. J Hosp Infect.

[22] Saha T, Murhekar M, Hutin YJ, Ramamurthy T (2009). An urban, water-borne outbreak of diarrhoea and shigellosis in a district town in eastern India. Natl Med J India.

[23] Oluyege JO, Dada AC, Odeyemi AT (2009). Incidence of multiple antibiotic resistant Gram-negative bacteria isolated from surface and underground water sources in south western region of Nigeria. Water Sci Technol.

[24] Blasi MF, Carere M, Pompa MG, Rizzuto E, Funari E (2008). Water-related diseases outbreaks reported in Italy. J Water Health.

[25] Kilic A, Li H, Mellmann A, Basustaoglu AC, Kul M, Senses Z (2008). *Acinetobacter septicus* sp. nov. association with a nosocomial outbreak of bacteremia in a neonatal intensive care unit. J Clin Microbiol.

[26] Toyoshima M, Chida K, Suda T (2010). Fulminant community-acquired pneumonia probably caused by *Acinetobacter lwoffii*. Respirology.

[27] Turton JF, Shah J, Ozongwu C, Pike R (2010). Incidence of *Acinetobacter* species other than *A. baumannii* among clinical isolates of *Acinetobacter*: evidence for emerging species. J Clin Microbiol.

[28] Regalado NG, Martin G, Antony SJ (2009). *Acinetobacter lwoffii*: bacteremia associated with acute gastroenteritis. Travel Med Infect Dis.

[29] Manfredi R, Nanetti A, Valentini R, Chiodo F (2001). [Pathogenic role of *Acinetobacter* spp during HIV infection]. Infez Med.

[30] Samra ZQ, Naseem M, Khan SJ, Dar N, Athar MA (2009). PCR targeting of antibiotic resistant bacteria in public drinking water of Lahore metropolitan, Pakistan. Biomed Environ Sci.

[31] Xi C, Zhang Y, Marrs CF, Ye W, Simon C, Foxman B (2009). Prevalence of antibiotic resistance in drinking water treatment and distribution systems. Appl Environ Microbiol.

[32] Wasfy MO, Oyofo BA, David JC, Ismail TF, El-Gendy AM, Mohran ZS (2000). Isolation and antibiotic susceptibility of *Salmonella, Shigella,* and *Campylobacter* from acute enteric infections in Egypt. J Health Popul Nutr.

[33] Mota MI, Gadea MP, González S, González G, Pardo L, Sirok A (2010). Bacterial pathogens associated with bloody diarrhea in Uruguayan children. Rev Argent Microbiol.

[34] Samie A, Guerrant RL, Barrett L, Bessong PO, Igumbor EO, Obi CL (2009). Prevalence of intestinal parasitic and bacterial pathogens in diarrhoeal and non-diarrhoeal human stools from Vhembe district, South Africa. J Health Popul Nutr.

[35] Pourakbari B, Mamishi S, Mashoori N, Mahboobi N, Ashtiani MH, Afsharpaiman S (2010). Frequency and antimicrobial susceptibility of *Shigella* species isolated in Children Medical Center Hospital, Tehran, Iran, 2001-2006. Braz J Infect Dis.

[36] Ram S, Vajpayee P, Shanker R (2008). Contamination of potable water distribution systems by multiantimicrobial-resistant enterohemorrhagic *Escherichia coli*. Environ Health Perspect.

[37] Shar AH, Kazi YF, Soomro IH (2009). Antibiotic susceptibility of thermo-tolerant *Escherichia coli* 2 isolated from drinking water of Khairpur City, Sindh, Pakistan. Pak J Biol Sci.

